# Application of Redox-Responsive Cysteine-Based Organogels as a Drug Delivery System for Doxorubicin

**DOI:** 10.1021/acsomega.4c02620

**Published:** 2024-12-25

**Authors:** Diba Zare, Gamze Yılmaz, Salih Özçubukçu

**Affiliations:** Department of Chemistry, Middle East Technical University, 06800 Ankara, Turkey

## Abstract

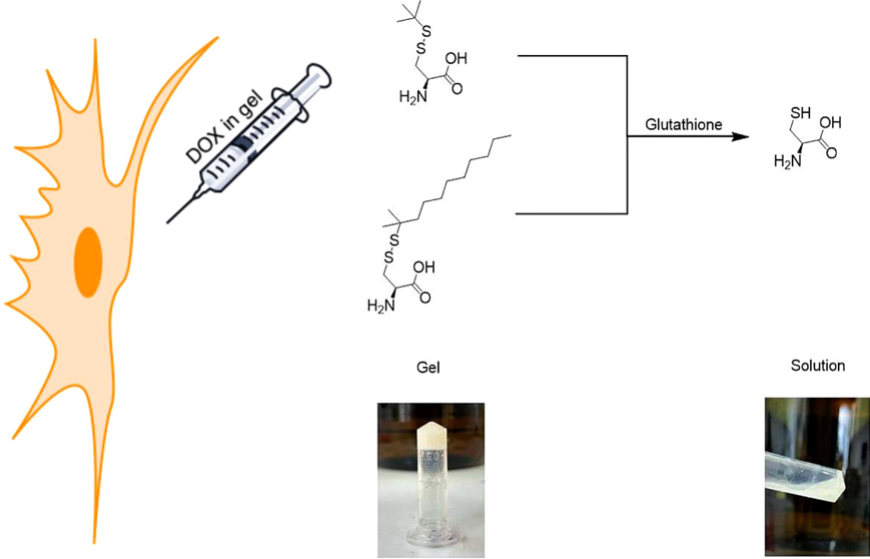

Cysteine derivatives having disulfide bonds in their side chains can be used as redox-responsive organogelators. The disulfide bond can be cleaved in the presence of certain reducing agents like thiol derivatives such as glutathione (GSH), which is a tripeptide that consists of cysteine, glutamic acid, and glycine. Studies show that cells of certain cancers have higher levels of glutathione due to increased production of reactive oxygen species (ROS). This feature allows targeted cancer therapy using glutathione-responsive drug delivery systems. This study showed the drug delivery property of l-Cys(*t*-dodecyl-sulfanyl)–OH and l-Cys-(S*t*Bu)–OH-based organogels. The drug-release properties of these organogels were measured in the presence of GSH and were compared with the drug-release property of the l-Cys-(*t*Bu)–OH-based organogel. The biocompatibility of the organogelators was measured *in vitro* by MTT assay and the characterization of microstructures and gel behaviors were studied using transmission electron microscopy (TEM) imaging, X-ray diffraction spectroscopy (XRD), Fourier-transform infrared spectroscopy (FTIR), and rheological measurements. The results indicated that the organogelators were able to form nanofibers by hydrogen bonds and van der Waals interactions between their hydrophobic groups and were able to release doxorubicin in the presence of GSH. The *in vitro* biocompatibility studies did not show significant toxicity to the L929 cells for l-Cys-(S*t*Bu)–OH and showed low concentrations of l-Cys(*t*-dodecyl-sulfanyl)–OH.

## Introduction

Cancer is one of the deadliest diseases and chemotherapy is its main clinical treatment even though most chemotherapeutic medicines, including doxorubicin, have poor water solubility, resulting in low bioavailability and significant side effects.^1,2^ Various methods are used to increase the solubility and bioavailability of chemotherapeutic drugs; among them, lipid-based formulations have shown promising results.^3,4^ Vegetable oil-based formulations can be used for controlled-release applications in the dosage forms of liquid, semisolid, or solid, which are known as emulsions, organogels, and microparticles, respectively.^5−8^ There has been much research into identifying and designing stimulus-sensitive agents for transporting and releasing chemotherapeutic drugs to the cancer area; in the past few decades, stimuli-responsive organogels have grabbed more attention as a potential drug delivery system for chemotherapy drugs.^9^

An organogel is a soft solid that contains both solid and liquid components, where the solid component, known as the gelator, is present as a network of aggregates that immobilize the nonpolar liquid component. Organogels have been synthesized using different gelators such as low-molecular-weight organogelators (LMWOs) that are based on organic compounds with less than 1 kDa molecular weights.^5,8^ Amino acids are biocompatible, versatile chemicals for self-assembled structures, and can easily be modified at the carboxyl or amine moieties and turn into numerous LMWOs.^8,10−12^ During the formation of organogels, the gelator molecules form a 3-D structure either by chemical cross-linking, like covalent and ionic bonds, or by physical entanglement, like hydrogen bonds, Van der Waal forces, and π–π interactions. The molecular packing of the organogels may be characterized by transmission electron microscopy (TEM), X-ray diffraction analysis (XRD), and Fourier-transform infrared (FTIR).^8,10,13^

Once a gel forms, changes in pH,^14^ redox environment,^15−17^ temperature,^14,18^ or exposure to certain enzymes or agents^19^ can lead to gel disruption, and this phenomenon has been used in drug-release studies. The functional groups in redox stimulus can undergo oxidation–reduction reactions, which have been associated with LMWOs to make the gel respond to the redox environment.^20^ Drug carriers containing disulfide bonds have been extensively studied because they are reductively cleavable in intracellular environments by the action of glutathione (GSH), a typical biological reducing agent.^15,16^ To maintain a highly reducing environment or enhanced oxidative stress, tumor cells are known to overproduce intracellular glutathione (GSH) or reactive oxygen species (ROS).^21,22^ The design and synthesis of intelligent biopolymer systems have been triggered by the thiolysis of the disulfide bond by GSH or the oxidation of the thioether to hydrophilic sulfoxide.^17,21^

In this study, cysteine derivatives having a disulfide bond in their side chain were used as a redox-responsive organogelator where the disulfide bond was cleaved in the presence of GSH. This feature allows targeted cancer therapy using glutathione-responsive drug delivery systems by the organogels’ form changing from the gel to solution state. The use of simple amino acid derivatives as low-molecular-weight organogelators is particularly novel because they offer biocompatibility and low toxicity, making them ideal for pharmaceutical applications. This aligns well with the goal of creating safer, more effective drug delivery systems that can specifically target cancer cells, ultimately minimizing the adverse effects on healthy tissues. The combination of low toxicity and targeted release represents a significant advancement in the development of organogels for medical applications. In our previous study,^10^ it has been shown that l-tyrosine having bulky side-chain protective groups is a good organogelator in a wide range of organic solvents. Therefore, l-cysteine with bulky side chains, such as l-Cys(*t*-dodecyl-sulfanyl)–OH (Long-SS) and l-Cys-(S*t*Bu)–OH (Short-SS), was chosen as organogelator in sunflower oil for use in drug delivery systems in the presence of GSH. l-Cys-(*t*Bu)–OH (No-SS) was also used for better comparison, as it did not contain a disulfide bond in its structure. The biocompatibility and morphological characterization of the obtained organogels was also carried out.

## Results and Discussion

### Synthesis of Organogelators and Their Gelation Properties

**Figure 1:**
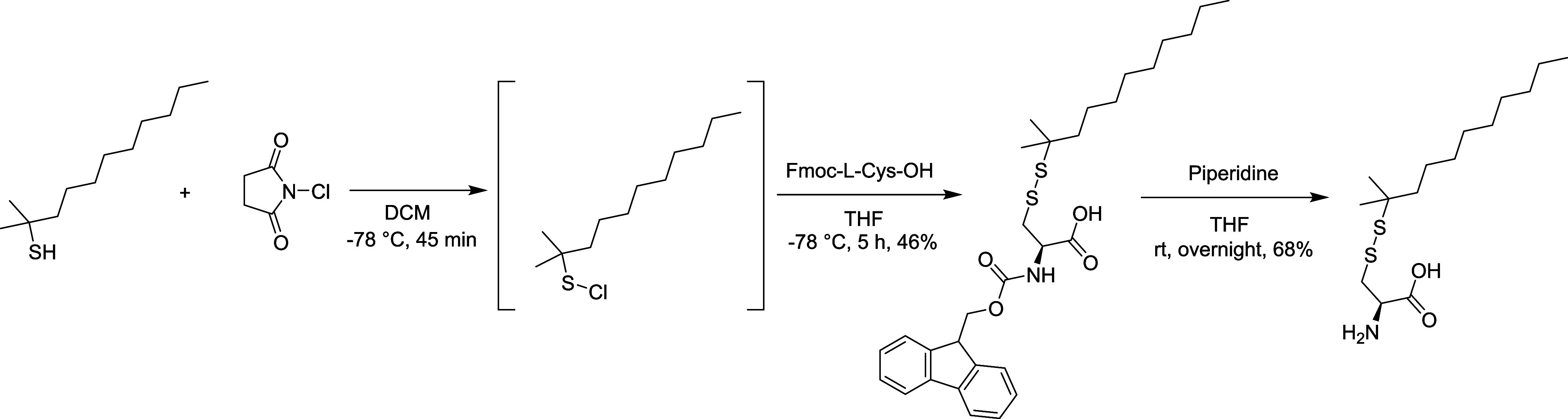
Synthesis of l-Cys(*t*-dodecyl-sulfanyl)–OH (Long-SS).

**Figure 2:**
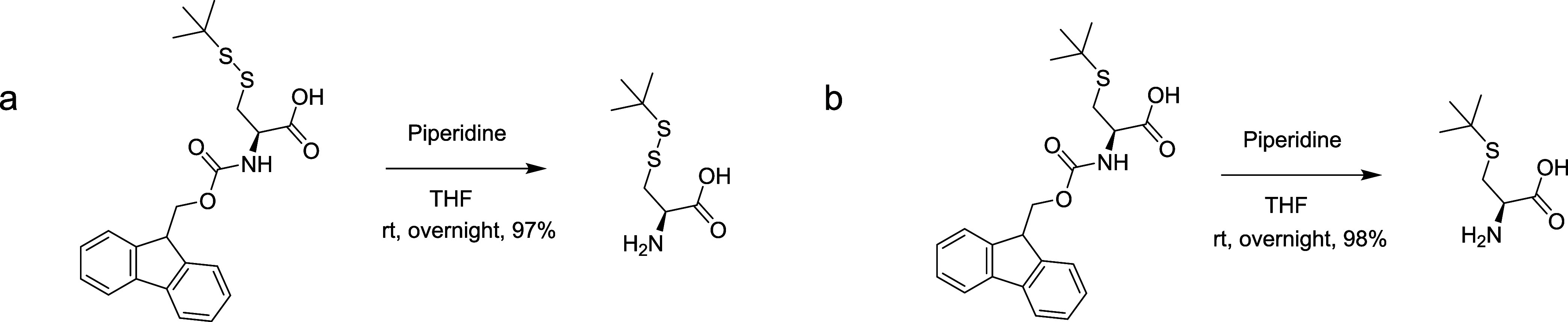
Synthesis of (a) l-Cys(StBu)–OH (Short-SS) and (b) l-Cys(*t*Bu)–OH (No-SS).

Fmoc-l-Cys(*t*-dodecyl-sulfanyl)–OH was synthesized by forming a disulfide bond between Fmoc-l-Cys-OH and *tert*-dodecanethiol using *N*-chlorosuccinimide (NCS) to increase its reactivity,^23^ followed by cleaving the Fmoc protecting group by piperidine to get l-Cys(*t*-dodecyl-sulfanyl)–OH (Long-SS) (Figure 1). l-Cys-(S*t*Bu)–OH (Short-SS) and l-Cys-(*t*Bu)–OH (No-SS) were synthesized by cleaving the Fmoc protecting group using piperidine from Fmoc-l-Cys(StBu)–OH and Fmoc-l-Cys(*t*Bu)–OH, respectively (Figure 2).

**Figure 3:**
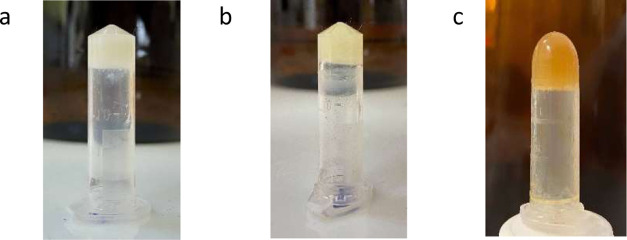
(a) Short-SS, (b) No-SS, and (c) Long-SS organogels.

To investigate the gelation properties of Long-SS, Short-SS, and No-SS, different combinations of organogelators, solvents, bases, sonication time, and gelation temperature were considered using the vial inversion method (Table 1).^10^ The results indicated that Short-SS was able to form a gel at 5 wt/v% in sunflower oil and THF at 35 °C, No-SS was able to form a gel at 6 wt/v% in sunflower oil and THF at 35 °C, and Long-SS was able to form a gel at 5 wt/v% in sunflower oil by adding 10 M NaOH(aq) as the base at 45 °C (Figure 3). NaOH allowed the resulting carboxylate group to participate better in hydrogen bond formation. Below these concentrations, temperature, and in the absence of 10 M NaOH(aq) for the Long-SS organogelator, gelation did not occur. Adding 10 M NaOH(aq) to Short-SS- and No-SS-based organogels was able to decrease the gelation percentage to 1% and sonication time to 5 min.

**Table 1:** Gelation Results of Short-SS, Long-SS, and No-SS Organogelators in Sunflower Oil

**organogelator**	**wt/v%**	**solvent**	**base**	**sonication time (min)**	**temperature (°C)**	**result**
Short-SS	5%	sunflower oil		20	35	gel
Long-SS	5%	sunflower oil	10 M NaOH(aq)	20	45	gel
No-SS	6%	sunflower oil		20	35	gel

### Characterization of Microstructures and Gel Behaviors

**Figure 4:**
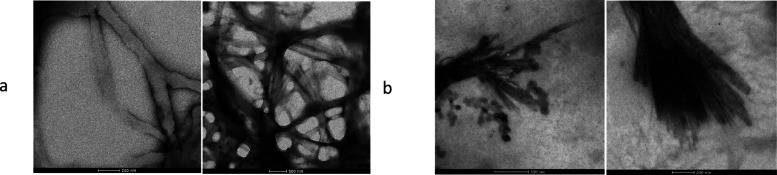
TEM images of the (a) 5 wt/v% Short-SS-based organogel in THF and (b) 5 wt/v% Long-SS-based organogel in sunflower oil.

To characterize the microstructures of organogels, transmission electron microscopy (TEM) imaging was performed, and to study the molecular packing at an atomic scale of organogels, powder X-ray diffraction (XRD) was performed. The TEM images of the 5 wt/v% Short-SS-based organogel in THF and 5 wt/v% Long-SS-based organogel in sunflower oil are shown in Figure 4. The TEM images of both Short-SS- and Long-SS-based organogels showed the formation of nanofibers with an approximate width of 120 nm.

**Figure 5:**
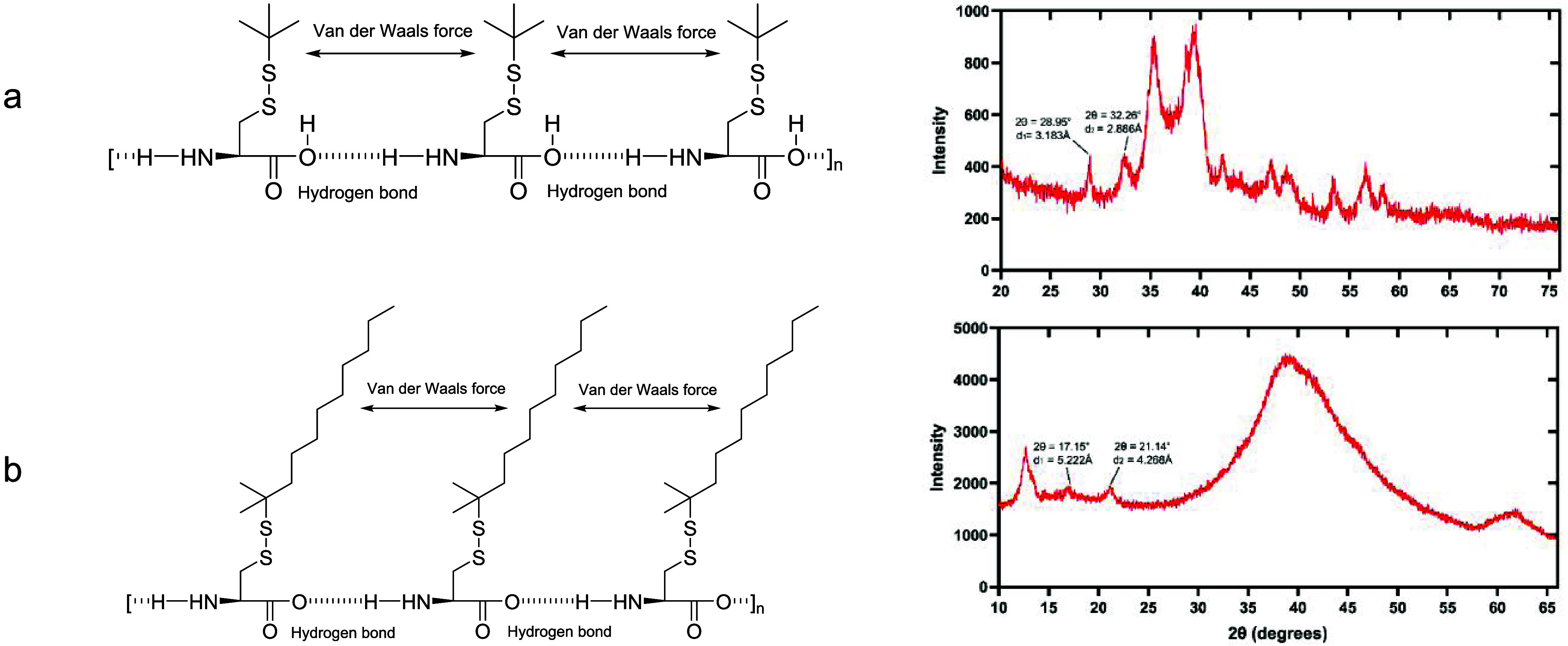
(a) Short-SS-based organogel structure and its XRD pattern; (b) long-SS-based organogel structure and its XRD pattern.

The physicochemical nature of the formulations was studied by X-ray diffraction (XRD) measurement to show the morphology of the formulations in their native state. Figure 5 shows the Short-SS- and Long-SS-based organogel structures and their XRD measurement. d_1_ represents the van der Waals interactions distance and d_2_ represents the hydrogen bonds distance of these organogels. The other peaks in the XRD pattern represent the atomic distances of the organogelators.

**Figure 6:**
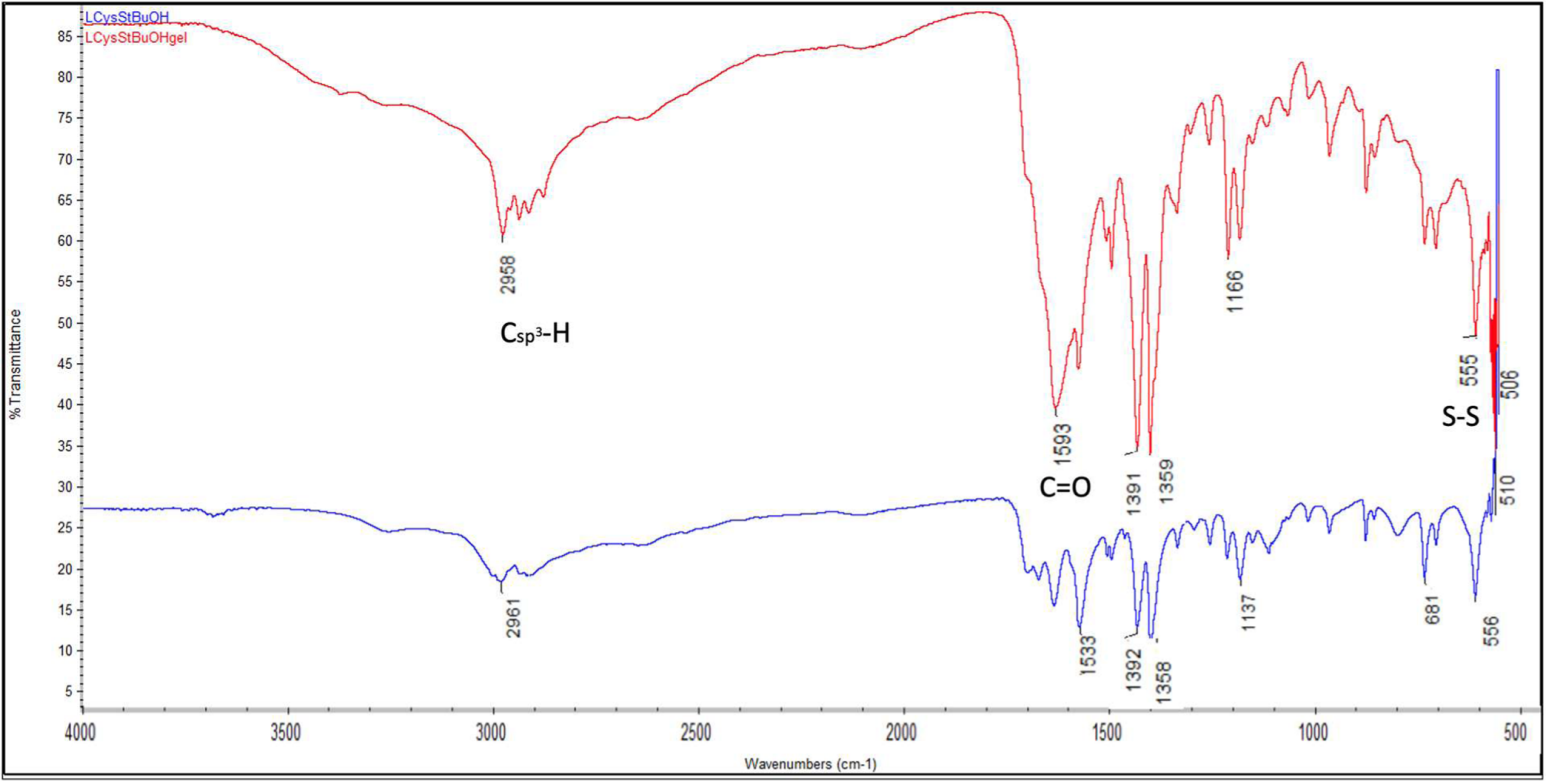
FTIR spectra of Short-SS gelator before (blue) and after (red) gelation.

The chemical interactions among the Short-SS organogelator before and after gelation were studied by Fourier-transform infrared (FTIR) spectroscopy (Figure 6). The broadening of bands around 1600 cm^–1^ after gelation indicates changes in the chemical interactions, possibly due to the involvement of carboxyl groups in the gelation process.

**Figure 7:**
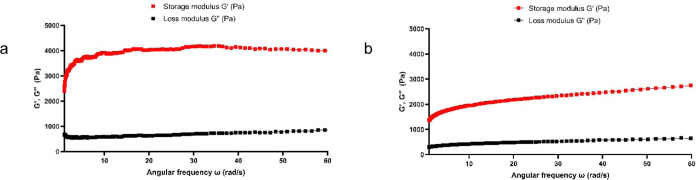
(a) Viscoelastic behavior of the Short-SS-based organogel and (b) viscoelastic behavior of the Long-SS-based organogel.

Gelation kinetics was determined by a time-sweep test within the linear viscoelastic range. Time-sweep tests of the 5 wt/v% Short-SS- and Long-SS-based organogels in sunflower oil were carried out until the storage and loss modulus reached a plateau. For both organogels, the storage modulus (*G*′) was greater than the loss modulus (*G*″), confirming the gel character of the resulting networks. The Short-SS-based organogel rheological graph represents a gel form and the Long-SS-based organogel rheological graph represents a paste-gel form (Figure 7).

### Drug-Release Kinetics of the Organogels

**Figure 8:**
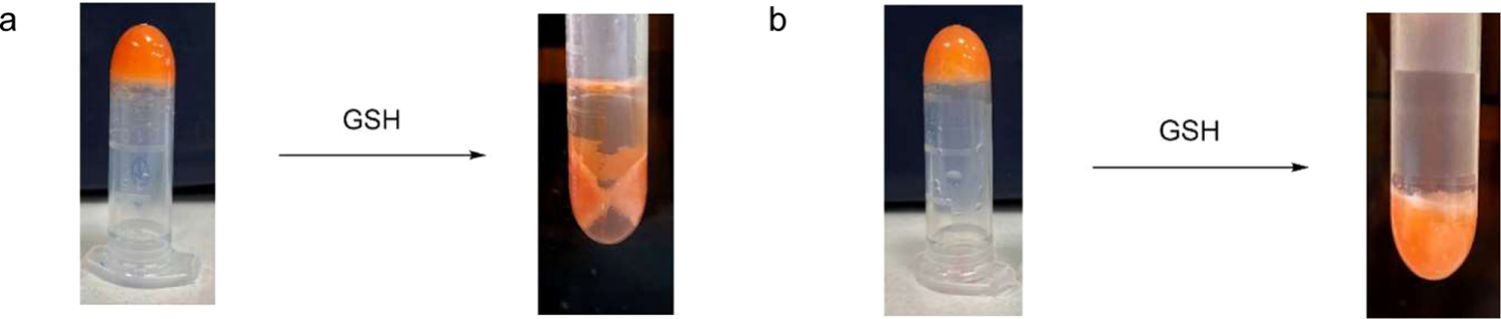
(a) Short-SS-based DOX-loaded organogel and (b) No-SS-based DOX-loaded organogel in the presence of GSH.

**Figure 9:**
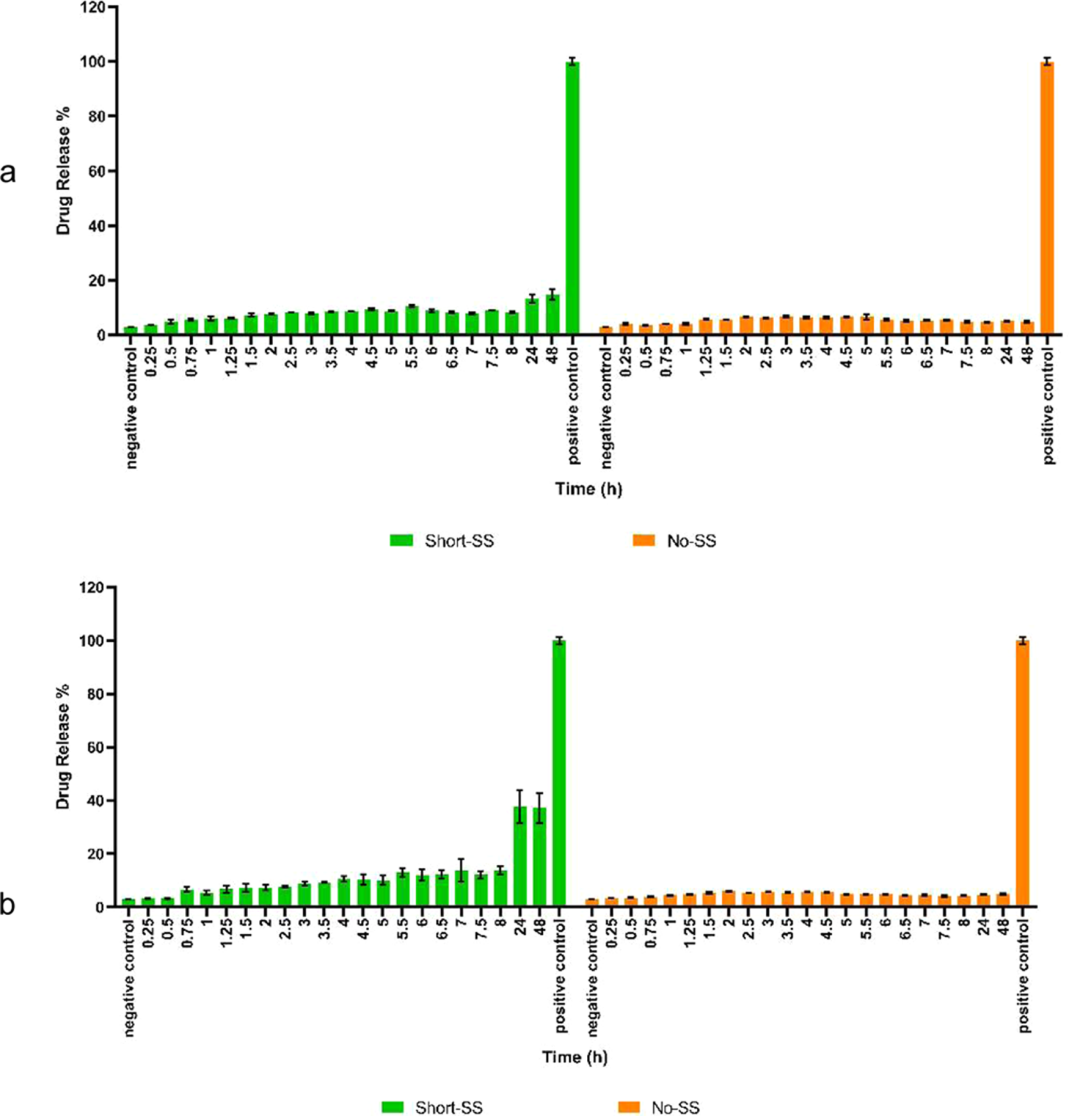
Drug-release kinetics of DOX-loaded 6 wt/v% Short-SS- and No-SS-based organogels in the presence of (a) low concentrations of GSH and (b) high concentrations of GSH.

**Figure 10:**
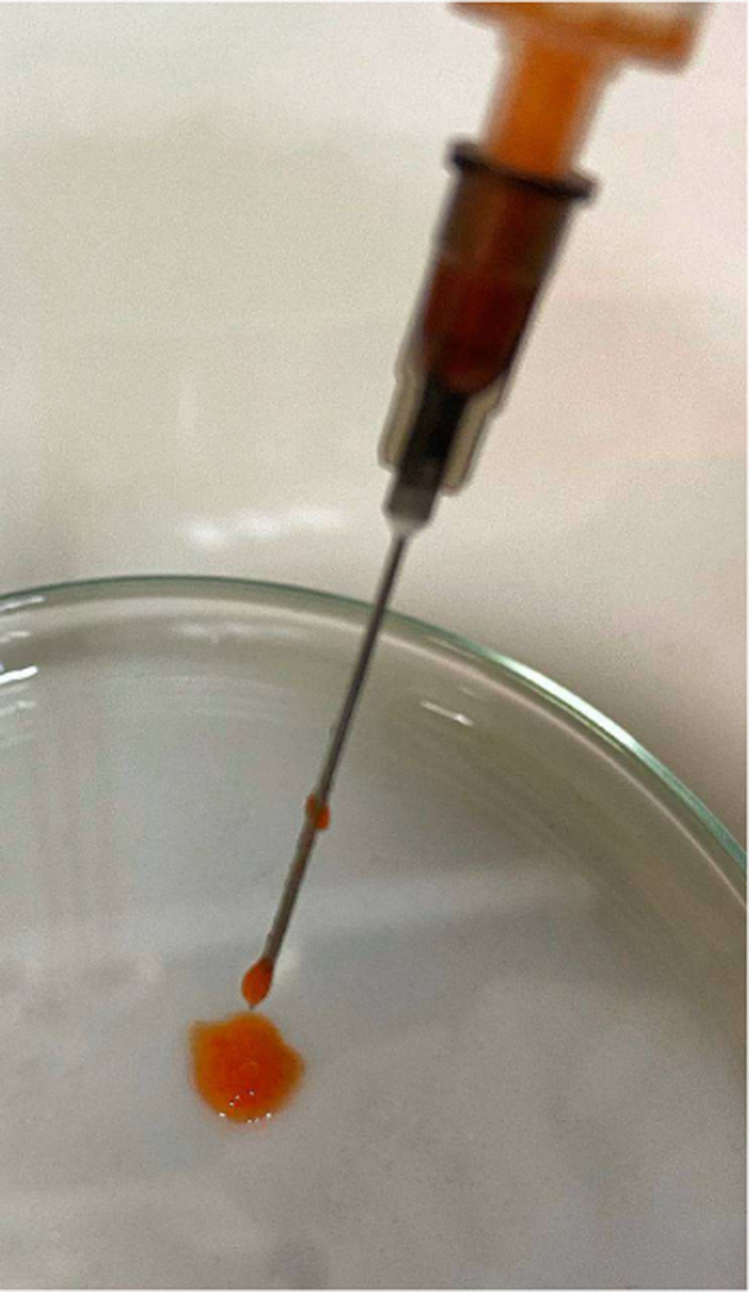
Injectability of Short-SS-based DOX-loaded organogel.

**Figure 11:**
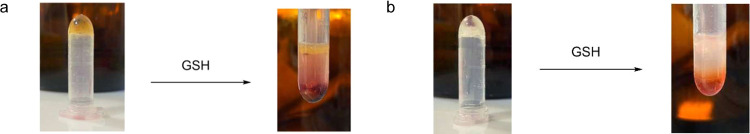
(a) Long-SS-based DOX-loaded organogel and (b) No-SS-based DOX-loaded organogel in the presence of GSH.

**Figure 12:**
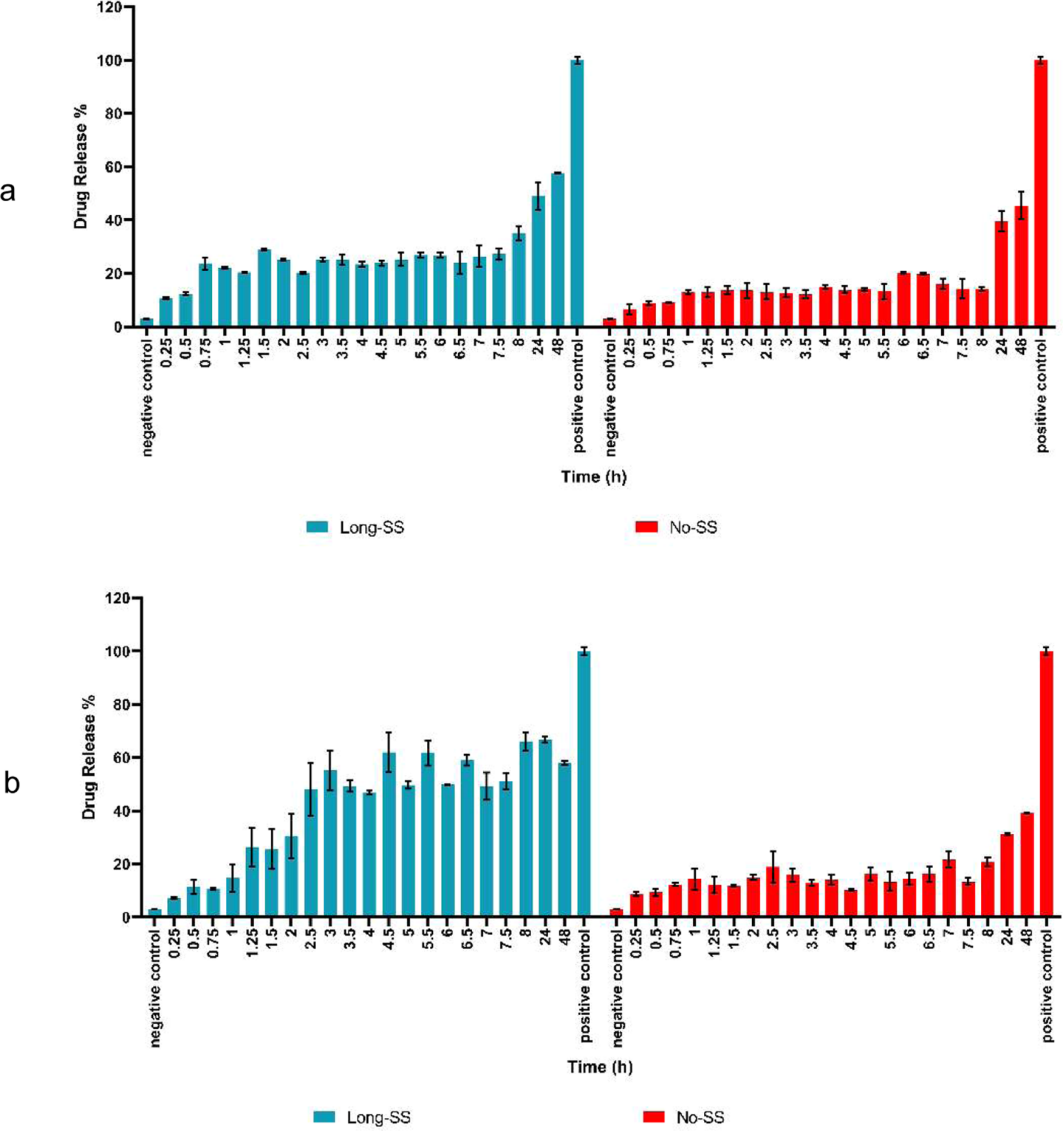
Drug-release kinetics of DOX-loaded 6 wt/v% Long-SS- and No-SS-based organogels in the presence of (a) low concentrations of GSH and (b) high concentrations of GSH.

In this study, we investigated the releasing time and amount of doxorubicin (DOX) from 6 wt/v% Short-SS- and 6 wt/v% Long-SS-based organogels network. The 6 wt/v% No-SS-based organogel was used as a control group as it does not contain a disulfide bond in its side chain. The experiment was carried out using two different concentrations of GSH, 8.68 mM (high) and 2.89 mM (low). Figure 8 shows the Short-SS-based DOX-loaded organogel turned into solution in the presence of GSH, while the No-SS-based DOX-loaded organogel remained in the gel form in the presence of GSH. As shown by the drug-release kinetic graphs of Short-SS-based organogels in the presence of high and low concentrations of GSH (Figure 9), the final amount of drug released from Short-SS-based organogels in the presence of high concentrations of GSH was approximately 2.5 times more than that at low concentrations of GSH, and both of them released more DOX than No-SS-based organogels. Moreover, the high concentration of GSH did not affect the No-SS-based organogels’ drug release as expected and remained at less than 5%. The drug-loading capacity of the Short-SS-based organogel shows that the doxorubicin in the propylene glycol solution amount cannot be more than 75% of the gel volume (Table 2). The injectability of the Short-SS-based DOX-loaded organogel is shown in Figure 10. Figure 11 shows the Long-SS-based DOX-loaded organogel turned into solution in the presence of GSH, while the No-SS-based DOX-loaded organogel remained in the gel form. As shown by the drug-release kinetic graphs of Long-SS-based organogels in the presence of high and low concentrations (Figure 12), the final amount of drug released from Long-SS-based organogels in the presence of high concentrations of GSH reached approximately 60%, 45 h earlier than that at low concentrations of GSH, and both of them released DOX more than No-SS-based organogels. Moreover, the high concentration of GSH did not affect the No-SS-based organogels’ drug release as expected and remained at approximately 40%.

**Table 2:** Drug-Loading Capacity of Short-SS-Based Organogel

**organogelator**	**wt/v%**	**solvent**	**0.01 g doxorubicin in 1.5 mL propylene glycol solution**	**result**
Short-SS	5%	0.2 mL sunflower oil	50 μL	gel
Short-SS	5%	0.2 mL sunflower oil	100 μL	gel
Short-SS	5%	0.2 mL sunflower oil	150 μL	gel
Short-SS	5%	0.2 mL sunflower oil	175 μL	solution
Short-SS	5%	0.2 mL sunflower oil	200 μL	solution

### *In Vitro* Biocompatibility Study

**Figure 13:**
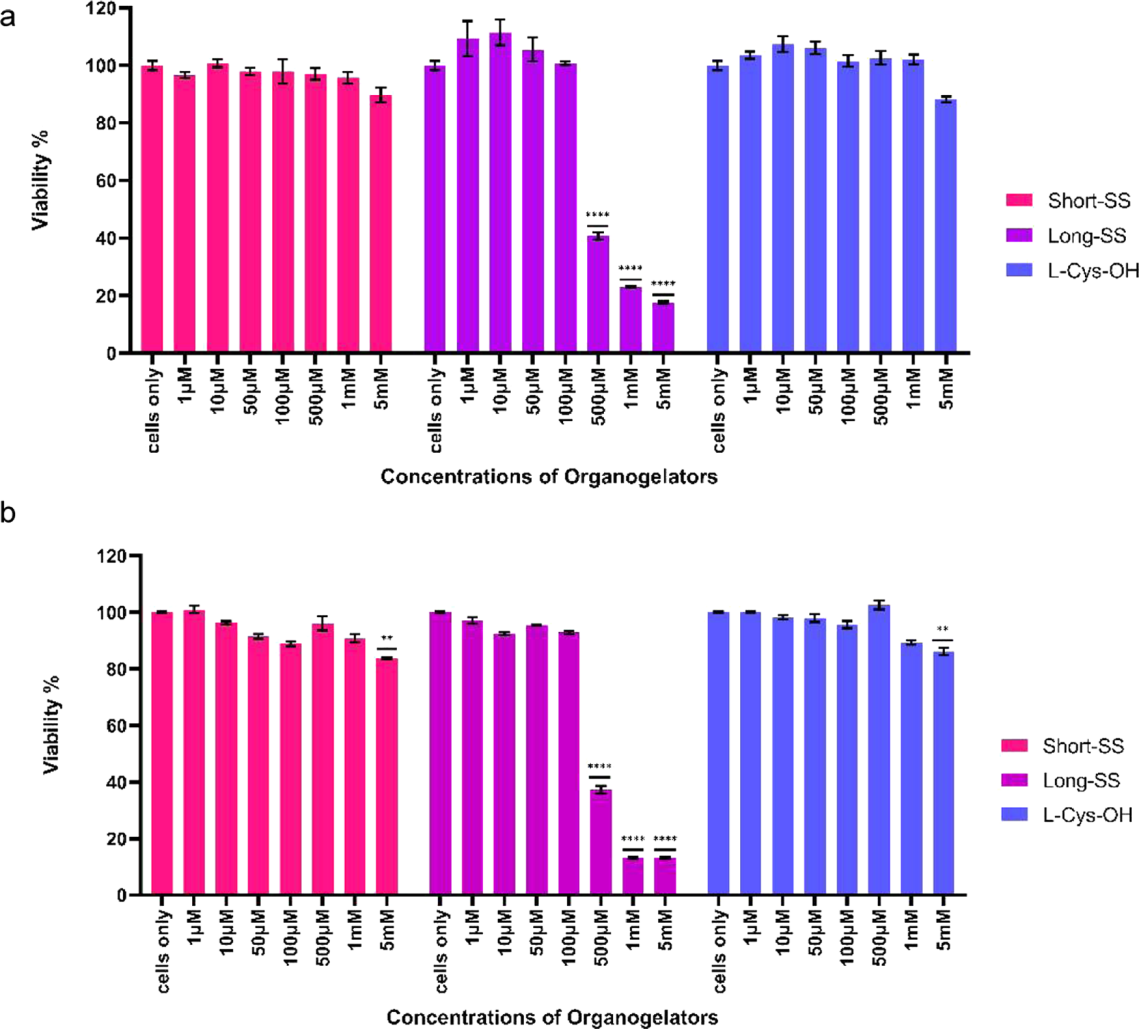
L929 cells viability treated with different concentrations of Short-SS, Long-SS, and l-Cys-OH for (a) 24 h incubation time and (b) 48 h incubation time.

To measure the toxicity of organogelators, we performed the MTT assay. L929 cells were treated with different concentrations of Long-SS and Short-SS organogelators, and for a better comparison of the organogelators’ toxicity, l-Cys-OH was used as a source of amino acid, from which these organogelators were derived. The results showed toxicity in the three highest concentrations (0.5–5 mM) of Long-SS and did not show toxicity for all concentrations of Short-SS and l-Cys-OH in 24 h incubation time. After 48-h incubation time, the highest concentration of Short-SS (5 mM), the three highest concentrations of Long-SS (0.5–5 mM), and the highest concentration of l-Cys-OH (5 mM) showed considerable toxicity to the cells (Figure 13).

## Conclusions

In conclusion, Short-SS is found to be one of the lightest low-molecular-weight organogelators in the literature (209 amu). It is also very easy to synthesize from commercially available simple cysteine derivatives. To the best of our knowledge, Short-SS and Long-SS are the only simple cysteine-derived organogelators that are stimuli responsive. The characterization studies confirmed the gel formation criteria and microfiber formation in both Short-SS- and Long-SS-based organogels, and the time-sweep test showed that Short-SS made a stiffer gel than the Long-SS organogelator. Although we expected the Long-SS to make a stiffer gel because of the possible stronger Van der Waals interactions between its long side chains, the *tert*-dodecanthiol that was used in this study was a mixture of isomers, so the difference in the side-chain structure of Long-SS molecules prevented the formation of strong Van der Waals interactions. Also, we had to add 10 M NaOH(aq) as the base to remove the hydrogen atom from Long-SS molecules to boost the hydrogen bond formation between them, and the temperature at which the Long-SS made the gel was higher than that of the Short-SS. The addition of 10 M NaOH(aq) as the base to the Long-SS-based gels caused the removal of the hydrogen atoms in the DOX structure and prevented the formation of a homogeneous DOX-loaded gel; thus, the DOX was injected into the middle of the gel after the gel formation and affected the drug-release studies. Therefore, it does not offer an efficient and predictable drug-release profile. Further research is required to find another method to coassemble with the drug to get a homogeneous drug-loaded gel. Moreover, the drug-release studies showed that GSH could turn the Short-SS- and Long-SS-based organogels into solution by cleaving the disulfide bond in their side chain; meanwhile, GSH did not affect the No-SS-based organogel as there is no disulfide bond in its side chain to be cleaved. Furthermore, the higher concentrations of GSH increased the amount of released DOX in Short-SS- and Long-SS-based organogels but did not affect the amount of released DOX in the No-SS-based organogels. The *in vitro* biocompatibility study showed significant toxicity for the three highest concentrations (0.5–5 mM) of Long-SS. Overall, the Short-SS-based organogel seems to be a better candidate for chemotherapy drug delivery applications in the form of an injectable organogel. Further chemical, *in vitro*, and *in vivo* studies should be performed to confirm its ability to be used as a drug delivery system.

## Materials and Methods

### Materials

Fmoc-l-Cys(StBu)–OH, Fmoc-l-Cys(*t*Bu)–OH, Fmoc-l-Cys(Trt)–OH, and l-Cys-OH·HCl were purchased from Chem-Impex International Inc. l-Glutathione reduced (GSH), phosphate-buffered saline (PBS), and triisopropylsilane (TIPS) were purchased from Sigma-Aldrich. Dichloromethane (DCM), tetrahydrofuran (THF), hexane, and trifluoroacetic acid (TFA) were purchased from Carlo Erba. *Tert*-dodecanethiol and *N*-chloro-succinimide (NCS) were purchased from Merck. Hydrochloric acid was purchased from Birpa. Diethyl ether (Et_2_O) and methanol were purchased from ISOLAB. 1,2-Propanediol purchased from Schuchardt München. Uranyl acetate was purchased from Fisher. DMEM was purchased from Serana. Piperidine was purchased from Thermo Scientific.

### Synthesis of Fmoc-l-Cys-OH

5.00 g (8.54 mmol) of Fmoc-l-Cys(Trt)–OH was dissolved in 340 mL of DCM, followed by adding 10 mL (48.8 mmol) of triisopropylsilane (TIPS) and 40 mL (0.52 mol) of trifluoroacetic acid (TFA). The reaction mixture was stirred for 10 min at room temperature and then concentrated under reduced pressure using Et_2_O as the co-solvent for removing TFA. The residue was suspended in hexane and centrifuged followed by discarding the supernatant, and the pellet was resuspended in hexane (cycle was repeated 5 times) until the complete removal of the trityl amino-protecting group. The pellet was dried under reduced pressure.^24^ 2.85 g (8.3 mmol) of white solid was obtained and the yield was 97%. TLC (DCM/MeOH = 10:1). The proton NMR spectrum is shown in Figure S2.

^1^H NMR (400 MHz, DMSO) δ 7.90 (d, *J* = 7.8 Hz, 2H), 7.74 (d, *J* = 7.5 Hz, 2H), 7.42 (t, *J* = 7.8 Hz, 2H), 7.34 (t, J = 7.8 Hz, 2H), 4.31 (d, *J* = 7.0 Hz, 2H), 4.25 (dd, *J* = 14.5, 8.0 Hz, 1H), 4.12 (td, *J* = 8.5, 4.3 Hz, 1H), 2.89 (ddd, *J* = 13.0, 8.4, 4.3 Hz, 1H), 2.73 (dt, *J* = 13.6, 8.5 Hz, 1H), 2.55 (brs, 1H).

### Synthesis of Fmoc-l-Cys(*t*-dodecyl-sulfanyl)–OH

2.4 g (18 mmol) of NCS (*N*-chlorosuccinimide) was dissolved in 66 mL of DCM at −78 °C and stirred at the same temperature for 20 min followed by adding 4.10 mL (17.4 mmol) of *tert*-dodecanethiol. The reaction was stirred at −78 °C temperature for another 45 min. The mixture was poured into a stirring solution of 3.00 g (8.7 mmol) of Fmoc-l-Cys-OH in 75 mL of THF at −78 °C. The whole mixture was stirred for the next 5 h, maintaining the temperature at −78 °C. The reaction was then allowed to come to room temperature (25 °C) and washed with acidified water (5% hydrochloric acid in water) 3 times. The organic layer was collected over MgSO_4_, filtered, and evaporated under reduced pressure. The crude was then purified by silica gel column chromatography using DCM/MeOH (10:1) as a mobile phase.^23^ 2.16 g (3.96 mmol) of a dark yellow viscous material was obtained and the yield was 46%. TLC (DCM/MeOH = 10:1). The proton and carbon NMR spectra are shown in Figures S4 and S5.

^1^H NMR (400 MHz, CDCl_3_) δ 7.69 (d, *J* = 7.6 Hz, 2H), 7.55 (d, *J* = 7.4 Hz, 2H), 7.33 (t, *J* = 7.5 Hz, 2H), 7.24 (t, *J* = 7.5 Hz, 2H), 4.76–4.60 (m, 1H), 4.40–4.29 (m, 2H), 4.18 (t, *J* = 7.0 Hz, 1H), 3.28–2.90 (m, 2H), 1.29–1.09 (m, 11H), 0.92–0.72 (m, 14 H). ^13^C NMR (100 MHz, CDCl_3_) δ: 175.0, 156.0, 143.6, 141.2, 127.5, 127.0, 125.1, 119.9, 67.4, 53.8, 46.9, 30.8, 29.6, 29.4, 29.3, 27.4, 26.6, 22.6, 14.3, 14.0, 12.2, 12.1, 8.7. HRMS C_30_H_41_NO_4_S_2_ [M + Na]^+^: calculated 566.2374 and found 566.2374 (Figure S6).

### Synthesis of l-Cys(*t*-dodecyl-sulfanyl)–OH

1.20 g (2.2 mmol) in 16 mL of THF and 1 mL of piperidine were stirred overnight at room temperature and then concentrated under reduced pressure. The residue was suspended in hexane and centrifuged, followed by discarding the supernatant, and the pellet was resuspended in hexane (cycle repeated 5 times) until the complete removal of the Fmoc protecting group. The pellet was dried under reduced pressure. 0.48 g (1.5 mmol) of a dark brown viscous material was obtained and the yield was 68%. TLC (DCM/MeOH = 10:1). The proton and carbon NMR spectra are shown in Figures S8 and S9.

^1^H NMR (400 MHz, CDCl_3_) δ 3.73–3.61 (m, 1H), 3.13–2.98 (m, 2H), 1.89–1.72 (m, 3H), 1.71–1.45 (m, 4H), 1.45–0.96 (m, 12H), 0.94–0.58 (m, 6H). ^13^C NMR (100 MHz, CDCl_3_) δ: 183.8, 68.1, 63.6, 50.5, 44.5, 38.7, 31.9 30.1, 29.7, 28.9, 22.5, 22.4, 14.1, 11.0. HRMS C_15_H_31_NO_2_S_2_ [M + H]^+^: calculated 322.1874, found 322.1699 (Figure S10).

### Synthesis of l-Cys(StBu)–OH

0.94 g (2.2 mmol) was dissolved in 16 mL of THF and 1 mL of piperidine and were stirred overnight at room temperature and then concentrated under reduced pressure, followed by washing with hexane using a filter paper until the complete removal of the Fmoc protecting group. 0.44 g (2.14 mmol) of a white solid was obtained and the yield was 97%. TLC (DCM/MeOH = 10:1). The proton NMR spectra are shown in Figure S12.

^1^H NMR (400 MHz, CD_3_OD) δ 3.73 (dd, *J* = 10.0, 3.4 Hz, 1H), 3.29 (dd, *J* = 14.1, 3.4 Hz, 1H), 2.86 (dd, *J* = 14.1, 10.1 Hz, 1H), 1.28 (s, 9H).

### Synthesis of l-Cys(*t*Bu)–OH

0.88 g (2.2 mmol) was dissolved in 16 mL of THF and 1 mL of piperidine and were stirred overnight at room temperature and then concentrated under reduced pressure, followed by washing with hexane using a filter paper until the complete removal of the Fmoc protecting group. 0.38 g (2.16 mmol) of a white solid was obtained and the yield was 98%. TLC (DCM/MeOH = 10:1). The proton NMR spectrum is shown in Figure S14.

^1^H NMR (400 MHz, CD_3_OD) δ 3.53 (dt, *J* = 9.7, 3.7 Hz, 1H), 3.14 (dd, *J* = 13.7, 3.6 Hz, 1H), 2.78 (dd, *J* = 13.7, 9.7 Hz, 1H), 1.27 (s, 9H).

### General Gelation Procedure

To prepare the 5 wt/v% l-Cys(StBu)–OH-based gel, 0.01 g of l-Cys(StBu)–OH was dissolved in 0.2 mL of the solvent in an Eppendorf tube followed by a 20 min ultrasonic bath at 35 °C. To prepare the 5 wt/v% l-Cys(*t*-dodecyl-sulfanyl)–OH-based gel, 0.01 g of l-Cys(*t*-dodecyl-sulfanyl)–OH was dissolved in 0.2 mL of the solvent in an Eppendorf tube, followed by adding 10 μL of 10 M NaOH(aq); the tube was placed for 20 min in an ultrasonic bath at 45 °C. To prepare the 6 wt/v% l-Cys(*t*Bu)–OH-based gel, 0.012 g of l-Cys(*t*Bu)–OH was dissolved in 0.2 mL of solvent in an Eppendorf tube followed by a 20 min ultrasonic bath at 35 °C. Gel formation occurred spontaneously. To test the gel formation, an inversion test was used.^10^

### Transmission Electron Microscopy Imaging

A 5 wt/v% l-Cys(StBu)–OH-based gel in THF and 5 wt/v% l-Cys(*t*-dodecyl-sulfanyl)–OH-based gel in sunflower oil were prepared and diluted 50-fold with THF and then applied on a Cu grid, and the excess solution was evaporated after 2 min, stained with 2% uranyl acetate solution for another 2 min, and washed with MiliQ water 2 times. An FEI Tecnai G2 Spirit BioTwin CTEM microscope was used to image the fibrillar formations after the self-assembly of the organogels.

### Powder X-ray Diffraction Measurements

The physicochemical nature of the formulations was studied by X-ray diffraction (XRD) measurement to show the morphology of the formulations in their native state. XRD measurements were conducted using X’Pert^3^ MRD with Cu Kα X-ray radiation (λ = 1.5406 Å). Five wt/v% l-Cys(StBu)–OH in THF was prepared and dried using a freeze-drier to obtain the xerogel. Five wt/v% l-Cys(*t*-dodecyl-sulfanyl)–OH in sunflower oil was prepared and diluted in THF and dried under reduced pressure to obtain the xerogel. The formulations were spread evenly over a glass plate and then subjected to an X-ray analysis. The distance of hydrogen and van der Waals bonds was calculated using the formula interplanar spacing (*d*) = order of reflection (*n*) × wavelength (λ)/2 × sin θ.

### Fourier-Transform Infrared Spectroscopic Measurements

The chemical interactions among the Short-SS organogelator before and after gelation were studied by FTIR measurements using Thermo Scientific Nicolet. Five wt/v% l-Cys(StBu)–OH in THF was prepared and dried using a freeze-drier to obtain the xerogel. l-Cys(StBu)–OH and the l-Cys(StBu)–OH-based xerogel were placed on top of the ATR sample holder and the IR light was directed through them.

### Rheological Measurements

Gelation kinetics of the l-Cys(StBu)–OH- and l-Cys(*t*-dodecyl-sulfanyl)–OH-based organogels were determined by a time-sweep test within the linear viscoelastic range using Physica MCR 301, Anton Paar. Five wt/v% l-Cys(StBu)–OH- and l-Cys(*t*-dodecyl-sulfanyl)–OH-based organogels were prepared in sunflower oil. The storage modulus (*G*′) and loss modulus (*G*″) were monitored under a strain sweep of 0.01–500% at a frequency of 10 rad/s at 25 °C.

### Drug-Release Kinetics

To measure the drug-release property of l-Cys(StBu)–OH-based organogels, 6 tubes of DOX-loaded 6 wt/v% l-Cys(StBu)–OH-based organogels and 6 tubes of DOX-loaded 6 wt/v% l-Cys(*t*Bu)–OH-based organogels in sunflower oil were prepared by adding 50 μL of 0.01 g of doxorubicin in 1.5 mL of propylene glycol solution into 0.012 g of organogelators and 0.2 mL of sunflower oil in Eppendorf tubes. The organogels were formed using a 35 °C sonic bath for 20 min and were stirred overnight. To measure the drug-release property of l-Cys(*t*-dodecyl-sulfanyl)–OH-based organogels, 6 tubes of DOX-loaded 6 wt/v% l-Cys(*t*-dodecyl-sulfanyl)–OH-based organogels and 6 tubes of DOX-loaded 6 wt/v% l-Cys(*t*Bu)–OH-based organogels in sunflower oil were prepared by adding 10 μL of 10 M NaOH(aq) to 0.012 g of organogelators and 0.2 mL of sunflower oil in Eppendorf tubes. The organogels were formed using a 45 °C sonic bath for 20 min for l-Cys(*t*-dodecyl-sulfanyl)–OH-based organogels and a 35 °C sonic bath for 5 min for l-Cys(*t*Bu)–OH-based organogels. The organogels were stirred overnight at room temperature followed by injecting 50 μL of 0.01 g of doxorubicin in 1.5 mL of propylene glycol solution in the middle of the gels and stirring overnight.

Organogels were divided into four groups: groups 1 and 2, each containing 3 tubes of DOX-loaded 6 wt/v% l-Cys(StBu)–OH-based organogel and 3 tubes of DOX-loaded 6 wt/v% l-Cys(*t*Bu)–OH-based organogel; groups 3 and 4, each containing 3 tubes of DOX-loaded 6 wt/v% l-Cys(*t*-dodecyl-sulfanyl)–OH-based organogel and 3 tubes of DOX-loaded 6 wt/v% l-Cys(*t*Bu)–OH-based organogel. One mL of 0.04 g of GSH in 45 mL of PBS with pH 7.2 (2.89 mM) was added on top of groups 1 and 3. One mL of 0.12 g of GSH in 45 mL of PBS with pH 7.2 (8.68 mM) was added on top of groups 2 and 4. 100 μL of the PBS-GSH solutions from the top of the gels was removed and replaced with 100 μL of fresh PBS-GSH solution every 15 min for the first 90 min and every 30 min for the next 6.5 h, followed by taking samples at 24 and 48 h. Free PBS was used as the negative control. 50 μL of 0.01 g of doxorubicin in 1.5 mL of propylene glycol was added into 1 mL of PBS and was used as the positive control. The absorbance of the released doxorubicin in the PBS solution on the top of the gels was measured at 480 nm using a BioTek epoch 2 microplate reader. Statistical analyses were performed using GraphPad Prism and the error bars indicate the standard error of the mean.

### *In Vitro* Biocompatibility Study

The biocompatibility of the organogelators was tested using the L929 cell line. 10 × 10^3^ cells were seeded in each well of the 96-well plates containing DMEM and 10% FBS and the cells were allowed to attach for 24 h. Ten mg of l-Cys(StBu)–OH, l-Cys(*t*-dodecyl-sulfanyl)–OH, and l-Cys-OH were sterilized under UV light for 20 min and were dissolved directly in 1 mL of the DMEM full medium as the main stock and were filtered using 0.22-μm filters. The cells were treated with l-Cys(StBu)–OH, l-Cys(*t*-dodecyl-sulfanyl)–OH, and l-Cys-OH with the final concentrations of 1 μM, 10 μM, 50 μM, 100 μM, 500 μM, 1 mM, and 5 mM in each well, and the negative control cells included untreated cells. The plates were incubated in a 5% CO_2_ incubator and maintained at 37 °C for 24 and 48 h. After 24 and 48 h of treatment, cells were incubated in the culture medium containing 10% MTT (3-(4,5-dimethylthiazol-2-yl)- 2,5-diphenyltetrazolium bromide) for 4 h, which resulted in the formation of formazan crystals. The crystals were solubilized in SDS overnight and then the absorbance of the dye was measured at 570 nm using the Thermo Scientific plate reader. Statistical analyses were performed using two-way ANOVA. The level of statistical significance is represented by ns for *p* > 0.05, * for *p* < 0.05, ** for *p* < 0.01, *** for *p* < 0.001, and **** for *p* < 0.0001. Error bars indicate the standard error of the mean.

## Supporting Information Available

Synthesis of Fmoc-l-Cys-OH; synthesis Fmoc-l-Cys(*t*-dodecyl-sulfanyl)-OH; synthesis of l-Cys(*t*-dodecyl-sulfanyl)-OH; synthesis of l-Cys(S*t*Bu)-OH; synthesis of l-Cys(*t*Bu)-OH (PDF)
