# Supplementary material for: Application of Redox-Responsive Cysteine-Based Organogels as a Drug Delivery System for Doxorubicin
Source: ACS Omega. 2024 Dec 25;10(1):147–56. doi: 10.1021/acsomega.4c02620 (PMC11740374; doi:10.1021/acsomega.4c02620)
Supplement: Supplementary file 1 — ao4c02620_si_001.pdf [file ao4c02620_si_001.pdf]

Application of Redox Responsive Cysteine-Based Organogels as a Drug Delivery System for Doxorubicin

Diba Zare[1], Gamze Yılmaz[1], & Salih Özçubukçu[1*]

[1]Middle East Technical University, Department of Chemistry, 06800, Ankara, Turkey.

Electronic Supporting Information (11 pages)

1. Materials………………. S2

2. Synthesis of Fmoc-L-Cys-OH ………….. ....……S2

3. Synthesis of Fmoc-L-Cys(*t*-dodecyl-sulfanyl)-OH S3

4. Synthesis of L-Cys(*t*-dodecyl-sulfanyl)-OH S6

5. Synthesis of L-Cys(S*t*Bu)-OH...S9

6. Synthesis of L-Cys(*t*Bu)-OH S10

Reeferences…… S11

1. Materials

Fmoc-L-Cys(S*t*Bu)-OH, Fmoc-L-Cys(*t*Bu)-OH, and Fmoc-L-Cys(Trt)-OH was purchased from Chem-Impex International Inc. Triisopropyl silane (TIPS) were purchased from Sigma-Aldrich. Dichloromethane (DCM), tetrahydrofuran (THF), hexane, and trifluoroacetic acid (TFA) were purchased from Carlo Erba. *Tert*-dodecanethiol and *N*-chloro-succinimide (NCS) were purchased from Merck Schuchardt. Hydrochloric acid was purchased from Birpa. Diethyl ether (Et_2O) and methanol purchased from ISOLAB. Piperidine was purchased from Thermo Scientific.

2. Synthesis of Fmoc-L-Cys-OH

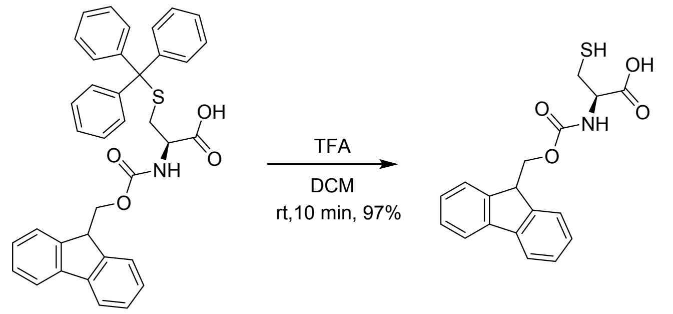

Figure S1: Synthesis of Fmoc-L-Cys-OH

5.00 g (8.54 mmol) Fmoc-L-Cys(Trt)-OH was dissolved in 340 mL DCM followed by adding 10 mL (48.8 mmol) triisopropylsilane (TIPS) and 40 mL (0.52 mol) trifluoroacetic acid (TFA), respectively. The reaction mixture was stirred for 10 minutes at room temperature and then concentrated under reduced pressure using Et_2O as the co-evaporated for removing TFA. The residue was suspended in hexane and centrifuged followed by discarding the supernatant and the pellet was resuspended in hexane (cycle was repeated 5 times) until the complete removal of the trityl amino protecting group. The pellet was dried under reduced pressure [1]. 2.85 g (8.3 mmol) of white solid was obtained and the yield was 97%. TLC (DCM: MeOH = 10: 1). The proton NMR is shown in Fig S2.

^{1}H NMR (400 MHz, DMSO) δ 7.90 (d, *J* = 7.8 Hz, 2H), 7.74 (d, *J* = 7.5 Hz, 2H), 7.42 (t, *J* = 7.8 Hz, 2H), 7.34 (t, J = 7.8 Hz, 2H), 4.31 (d, *J* = 7.0 Hz, 2H), 4.25 (dd, *J* = 14.5, 8.0 Hz, 1H), 4.12 (td, *J* = 8.5, 4.3 Hz, 1H), 2.89 (ddd, *J* = 13.0, 8.4, 4.3 Hz, 1H), 2.73 (dt, J = 13.6, 8.5 Hz, 1H), 2.55 (brs, 1H).

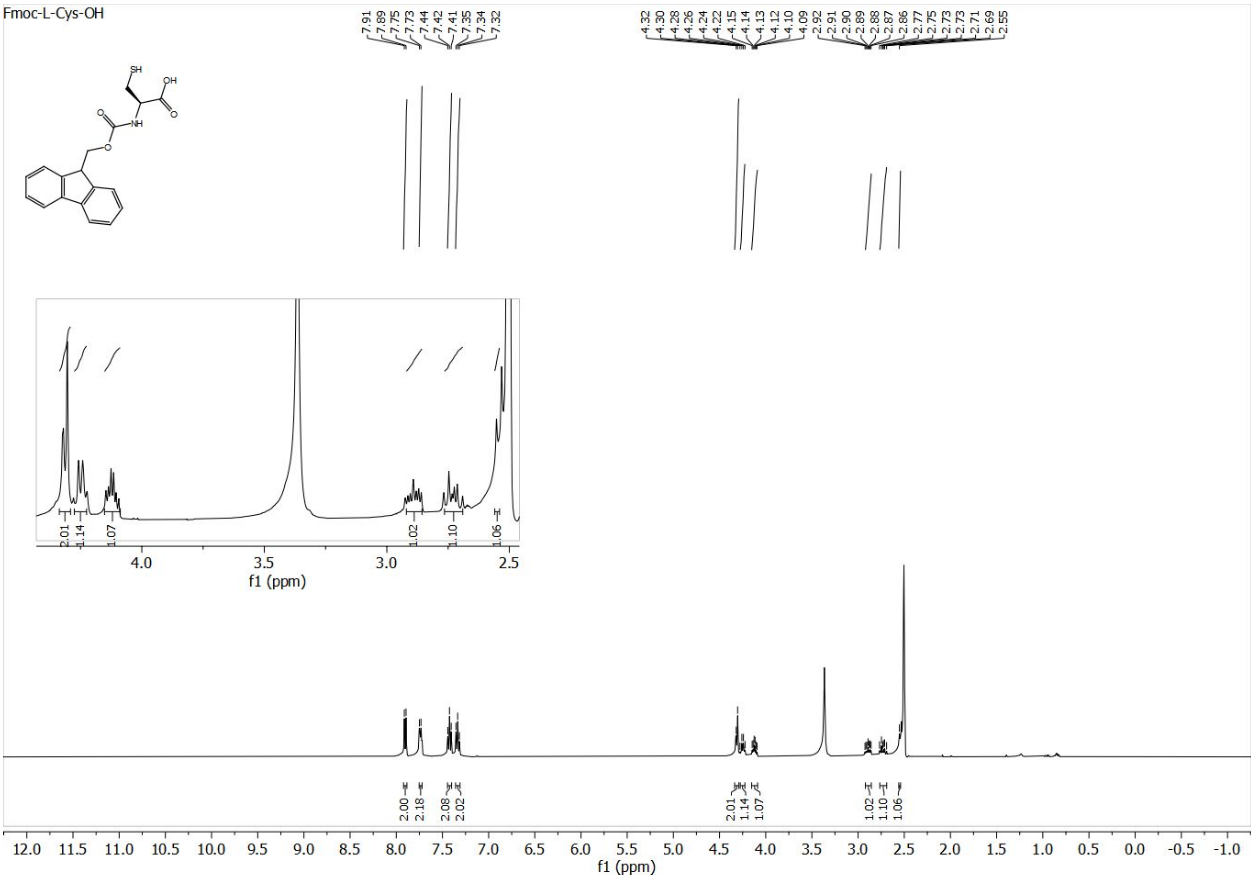

Figure S2: ^{1}H NMR spectrum of Fmoc-L-Cys-OH (400 MHz, DMSO).

3. Synthesis of Fmoc-L-Cys(*t*-dodecyl-sulfanyl)-OH

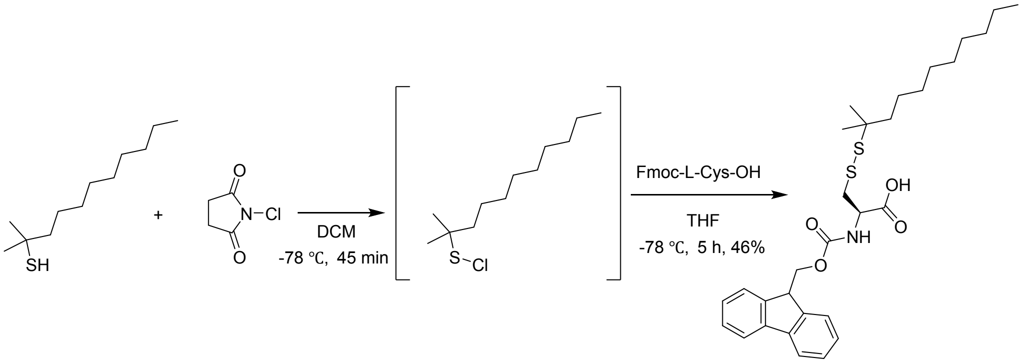

Figure S3: Synthesis of Fmoc-L-Cys(*t*-dodecyl-sulfanyl)-OH

2.4 g (18 mmol) NCS (N-chlorosuccinimide) was dissolved in 66 mL DCM at -78 °C and stirred at the same temperature for 20 minutes followed by adding 4.10 mL (17.4 mmol) *tert*-dodecanethiol. The reaction was stirred at -78 °C temperature for another 45 minutes. The mixture was poured into a stirring solution of 3.00 g (8.7 mmol) Fmoc-L-Cys-OH in 75 mL THF at -78 °C. The whole mixture was stirred for the next 5 hours

maintaining the temperature at -78 °C. The reaction was then allowed to come to room temperature (25 °C) and washed with acidified water (5% hydrochloric acid in water) 3 times. The organic layer was collected over $MgSO_4$, filtered, and evaporated under reduced pressure. The crude was then purified using silica gel column chromatography, using DCM: MeOH (10: 1) as a mobile phase [2]. 2.16 g (3.96 mmol) of dark yellow viscose material was obtained and the yield was 46%. TLC (DCM: MeOH = 10: 1). The proton and carbon NMR are shown in Fig S4 and Fig S5.

^{1}H NMR (400 MHz, $CDCl_3$) δ 7.69 (d, J = 7.6 Hz, 2H), 7.55 (d, J = 7.4 Hz, 2H), 7.33 (t, J = 7.5 Hz, 2H), 7.24 (t, J = 7.5 Hz, 2H), 4.76 – 4.60 (m, 1H), 4.40 – 4.29 (m, 2H) 4.18 (t, J = 7.0 Hz, 1H) 3.28 – 2.90 (m, 2H), 1.29 – 1.09 (m, 11H), 0.92 – 0.72 (m, 14 H). ^{13}C NMR (100 MHz, $CDCl_3$) δ 175.0, 156.0, 143.6, 141.2, 127.6, 127.0, 125.1, 119.9, 67.4, 53.8, 46.9, 30.8, 29.6, 29.4, 29.3, 27.4, 26.6, 22.6, 14.3, 14.0, 12.2, 12.1, 8.7. HRMS $C_{30}H_{41}NO_4S_2$ $[M+Na]^+$: Calculated 566.2374, found 566.2374 (Fig S6).

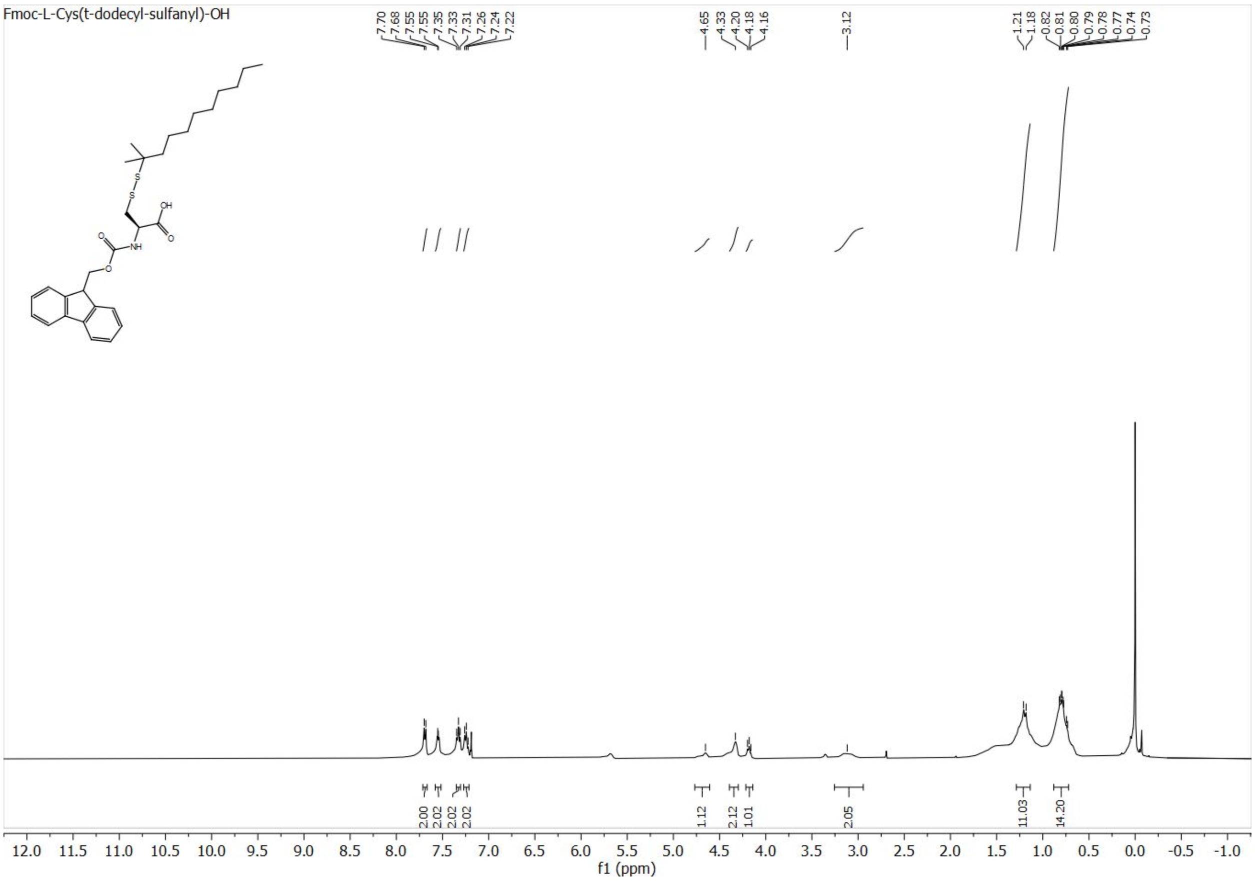

Figure S4: ^{1}H NMR spectrum of Fmoc-L-Cys(t-dodecyl-sulfanyl)-OH (400 MHz $CDCl_3$).

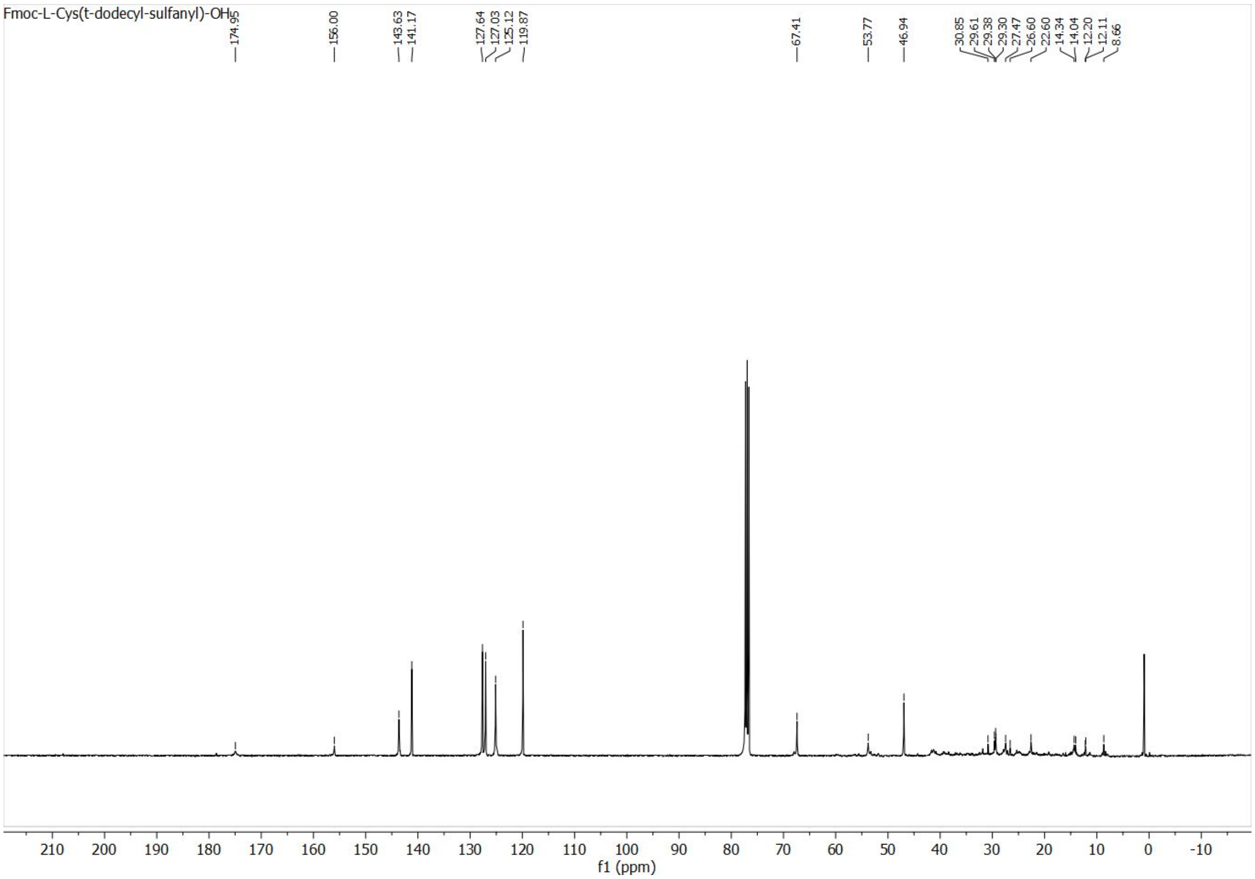

Figure S5: ^{13}C NMR spectrum of Fmoc-L-Cys(*t*-dodecyl-sulfanyl)-OH (100 MHz $CDCl_3$).

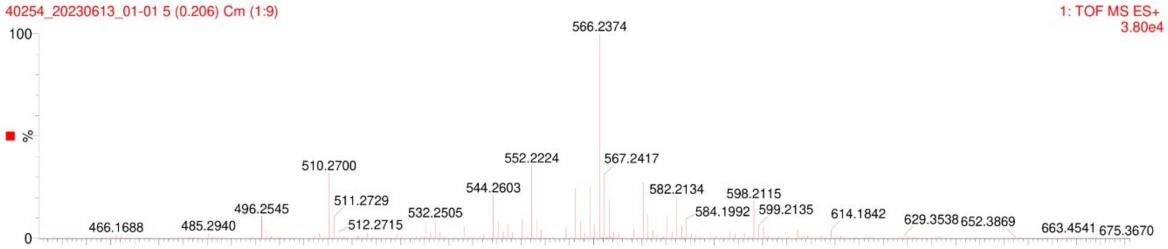

Figure S6: HRMS chromatogram of Fmoc-L-Cys(*t*-dodecyl-sulfanyl)-OH.

4. Synthesis of L-Cys(*t*-dodecyl-sulfanyl)-OH

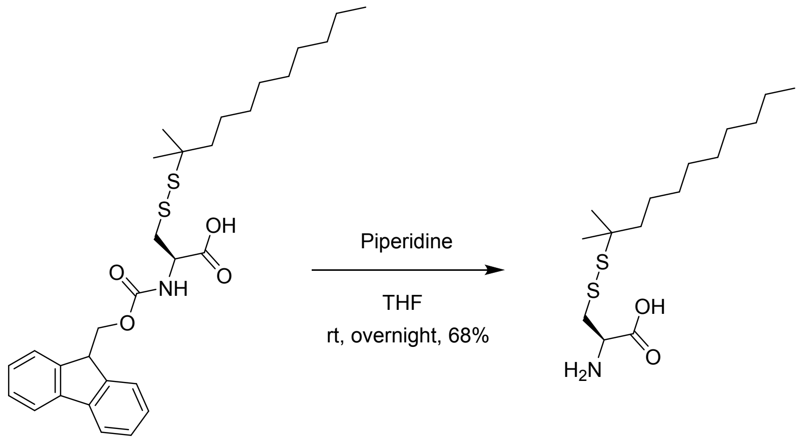

Figure S7: Synthesis of L-Cys(*t*-dodecyl-sulfanyl)-OH

1.20 g (2.2 mmol) Fmoc-L-Cys(*t*-dodecyl-sulfanyl)-OH in 16 mL THF and 1 mL piperidine was stirred overnight at room temperature and then concentrated under reduced pressure. The residue was suspended in hexane and centrifuged followed by discarding the supernatant and the pellet was resuspended in hexane (cycle repeated 5 times) until the complete removal of the Fmoc protecting group. The pellet was dried under reduced pressure. 0.48 g (1.5 mmol) of dark brown viscose material was obtained, and the yield was 68%. TLC (DCM: MeOH = 10: 1) The proton and carbon NMR are shown in Fig S8 and Fig S9.

^{1}H NMR (400 MHz, $CDCl_3$) δ 3.73-3.61 (m, 1H), 3.13-2.98 (m, 2H), 1.89-1.72 (m, 3H), 1.71-1.45 (m, 4H), 1.45-0.96 (m, 12H), 0.94-0.58 (m, 6H). ^{13}C NMR (100 MHz, $CDCl_3$) δ 183.8, 68.1, 63.6, 50.5, 44.5, 38.7, 31.9 30.1, 29.7, 28.9, 22.5, 22.4, 14.1, 11.0. HRMS $C_{15}H_{31}NO_2S_2$ $[M+H]^+$: Calculated 322.1874, found 322.1699 (Fig S10).

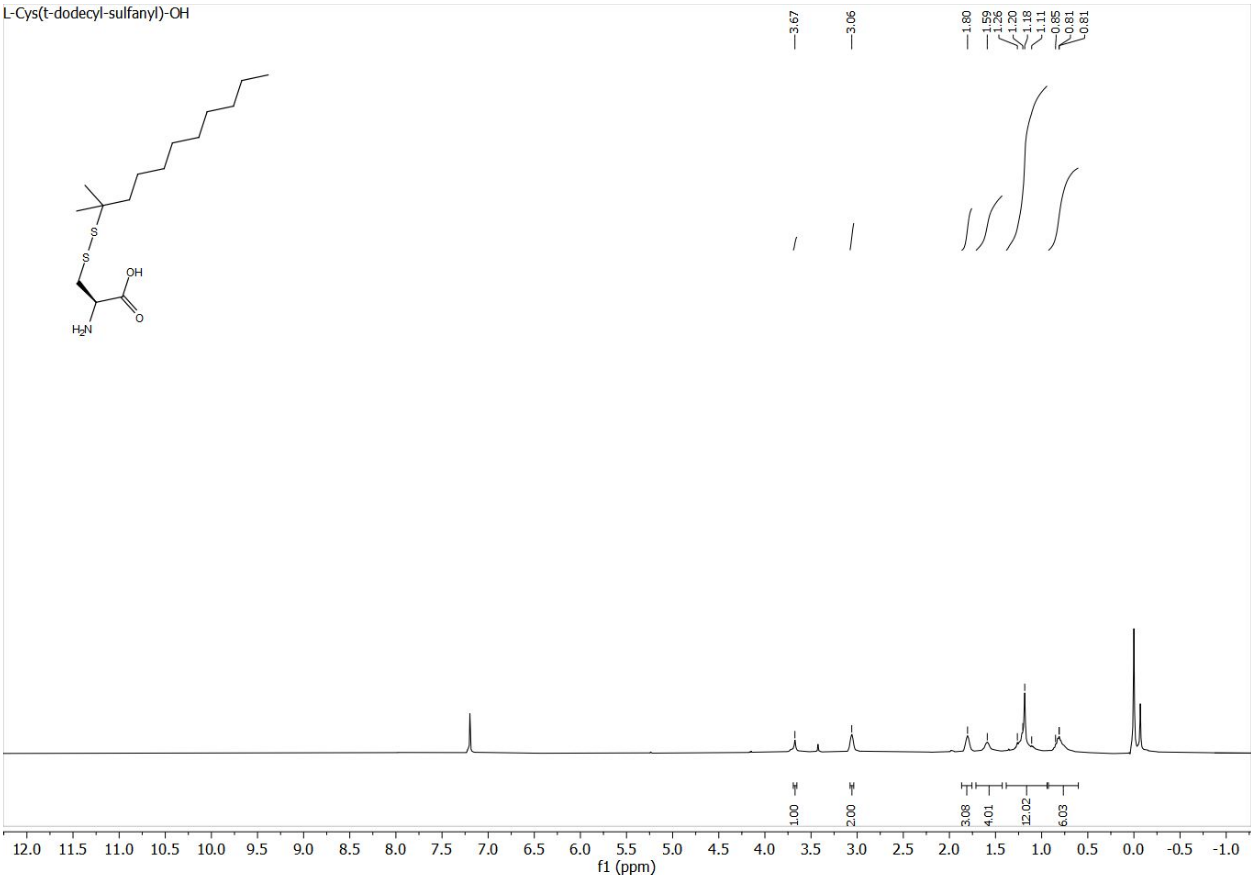

Figure S8: ^{1}H NMR spectrum of L-Cys(*t*-dodecyl-sulfanyl)-OH (400 MHz $CDCl_3$).

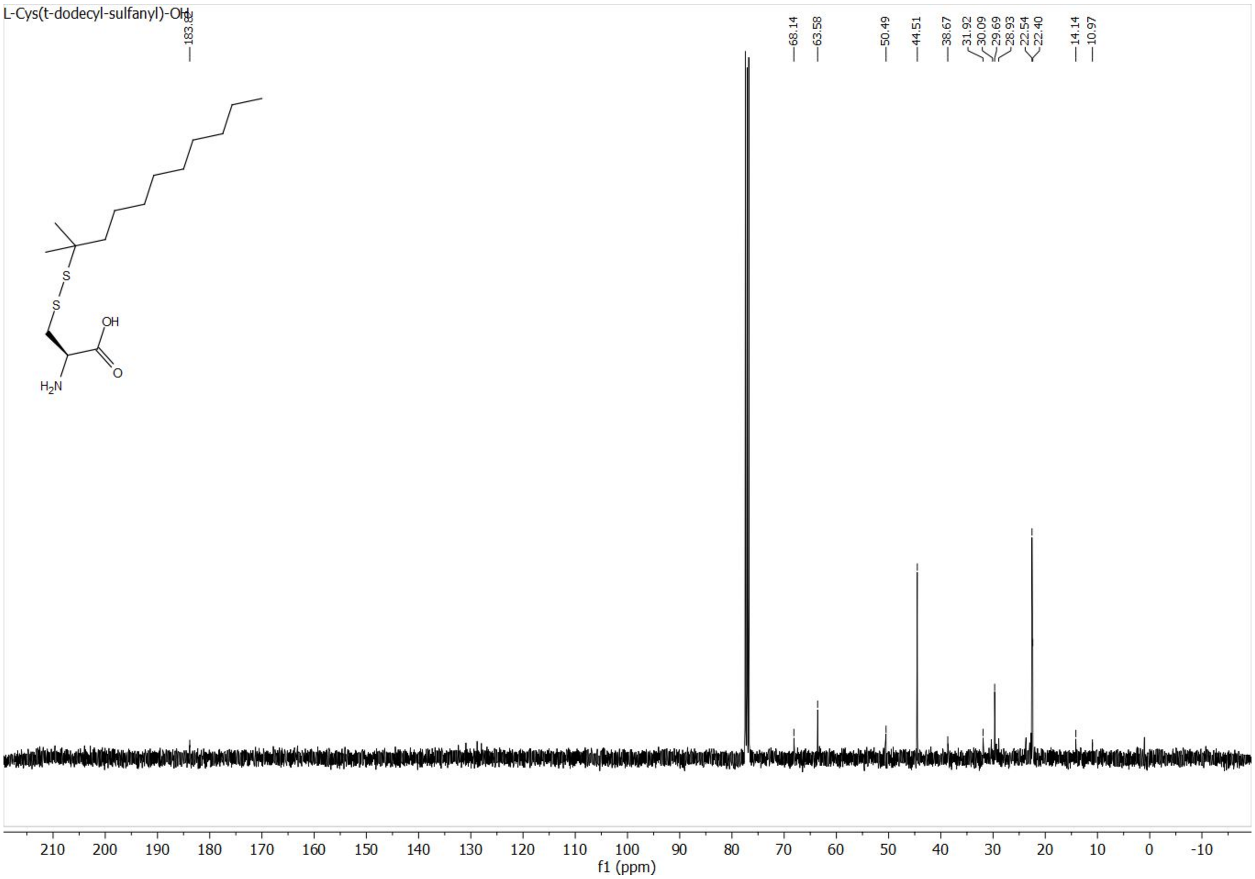

Figure S9: ^{13}C NMR spectrum of L-Cys(*t*-dodecyl-sulfanyl)-OH (100 MHz $CDCl_3$).

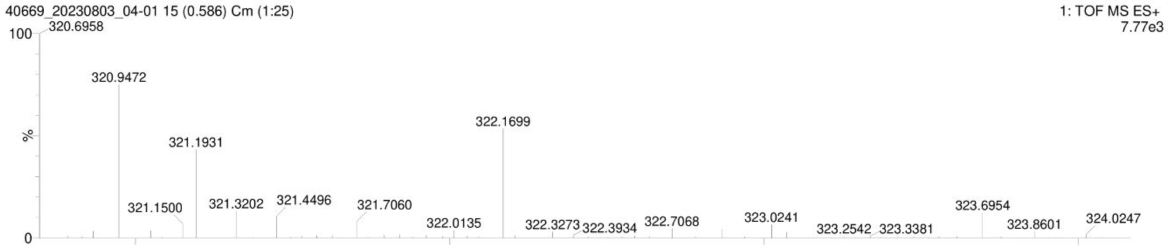

Figure S10: HRMS chromatogram of L-Cys(*t*-dodecyl-sulfanyl)-OH.

5. Synthesis of L-Cys(S*t*Bu)-OH

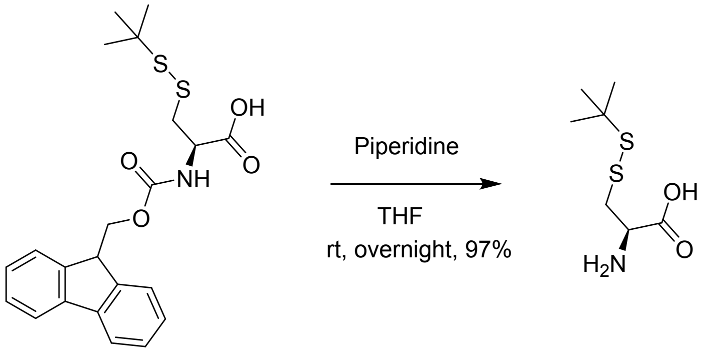

Figure S11: Synthesis of L-Cys(S*t*Bu)-OH

0.94 g (2.2 mmol) Fmoc-L-Cys(S*t*Bu)-OH was dissolved in 16 mL THF and 1 mL piperidine and was stirred overnight at room temperature and then concentrated under reduced pressure followed by washing with hexane using a filter paper until the complete removal of the Fmoc protecting group. 0.44 g (2.14 mmol) of white solid was obtained and the yield was 97%. TLC (DCM: MeOH = 10: 1) The proton NMR is shown in Fig S12.

^{1}H NMR (400 MHz, CD_3OD) δ 3.73 (dd, *J* = 10.0, 3.4 Hz, 1H), 3.29 (dd, *J* = 14.1, 3.4 Hz, 1H), 2.86 (dd, *J* = 14.1, 10.1 Hz, 1H), 1.28 (s, 9H).

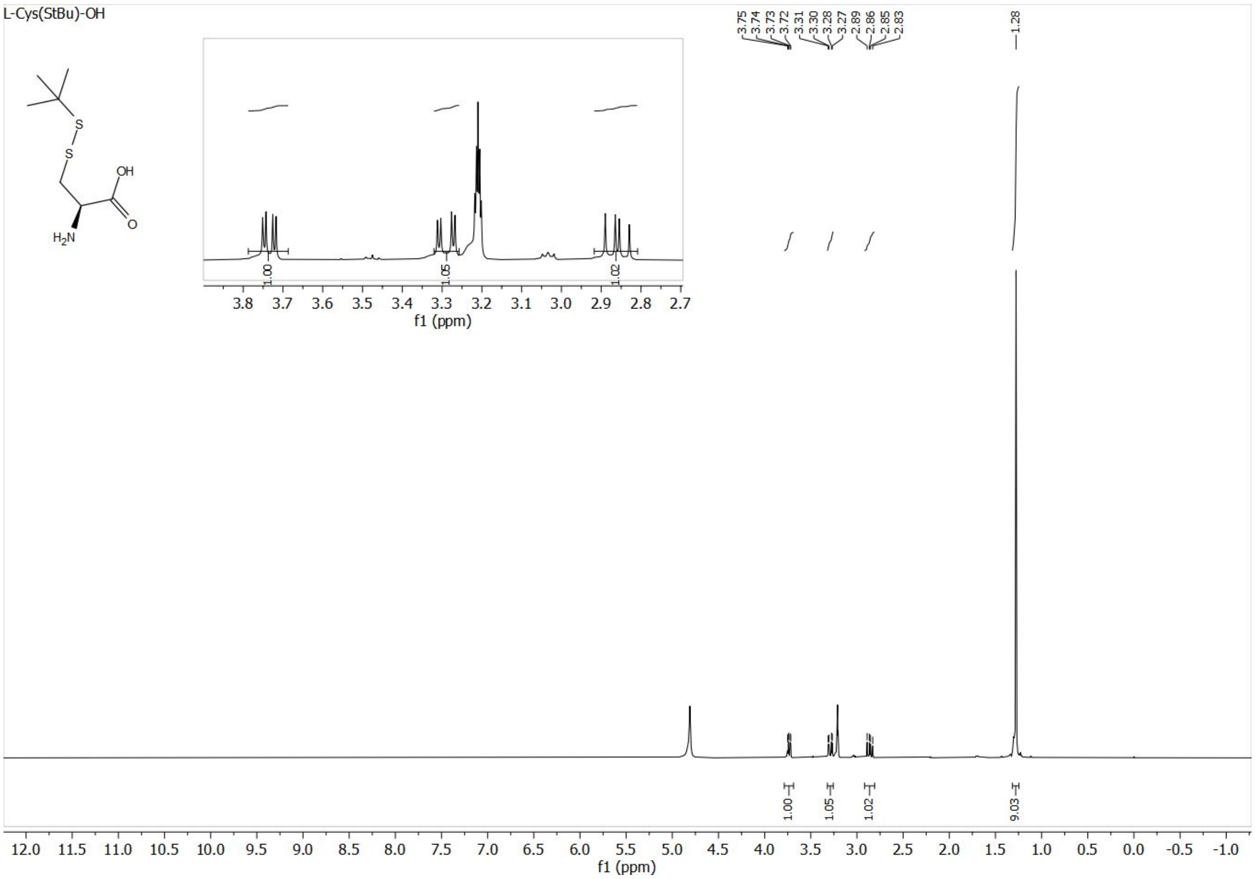

Figure S12: ^{1}H NMR spectrum of L-Cys(S*t*Bu)-OH (400 MHz CD_3OD).

6. Synthesis of L-Cys(*t*Bu)-OH

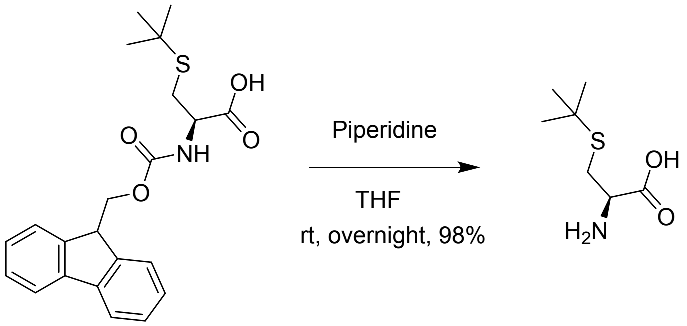

Figure S13: Synthesis of L-Cys(*t*Bu)-OH

0.88 g (2.2 mmol) Fmoc-L-Cys(*t*Bu)-OH was dissolved in 16 mL THF and 1 mL piperidine and was stirred overnight at room temperature and then concentrated under reduced pressure followed by washing with hexane using a filter paper until the complete removal of the Fmoc protecting group. 0.38 g (2.16 mmol) of

white solid was obtained and the yield was 98%. TLC (DCM: MeOH = 10: 1) The proton NMR is shown in Fig S14.

^{1}H NMR (400 MHz, CD_3OD) δ 3.53 (dt, *J* = 9.7, 3.7 Hz, 1H), 3.14 (dd, *J* = 13.7, 3.6 Hz, 1H), 2.78 (dd, *J* = 13.7, 9.7 Hz, 1H), 1.27 (s, 9H).

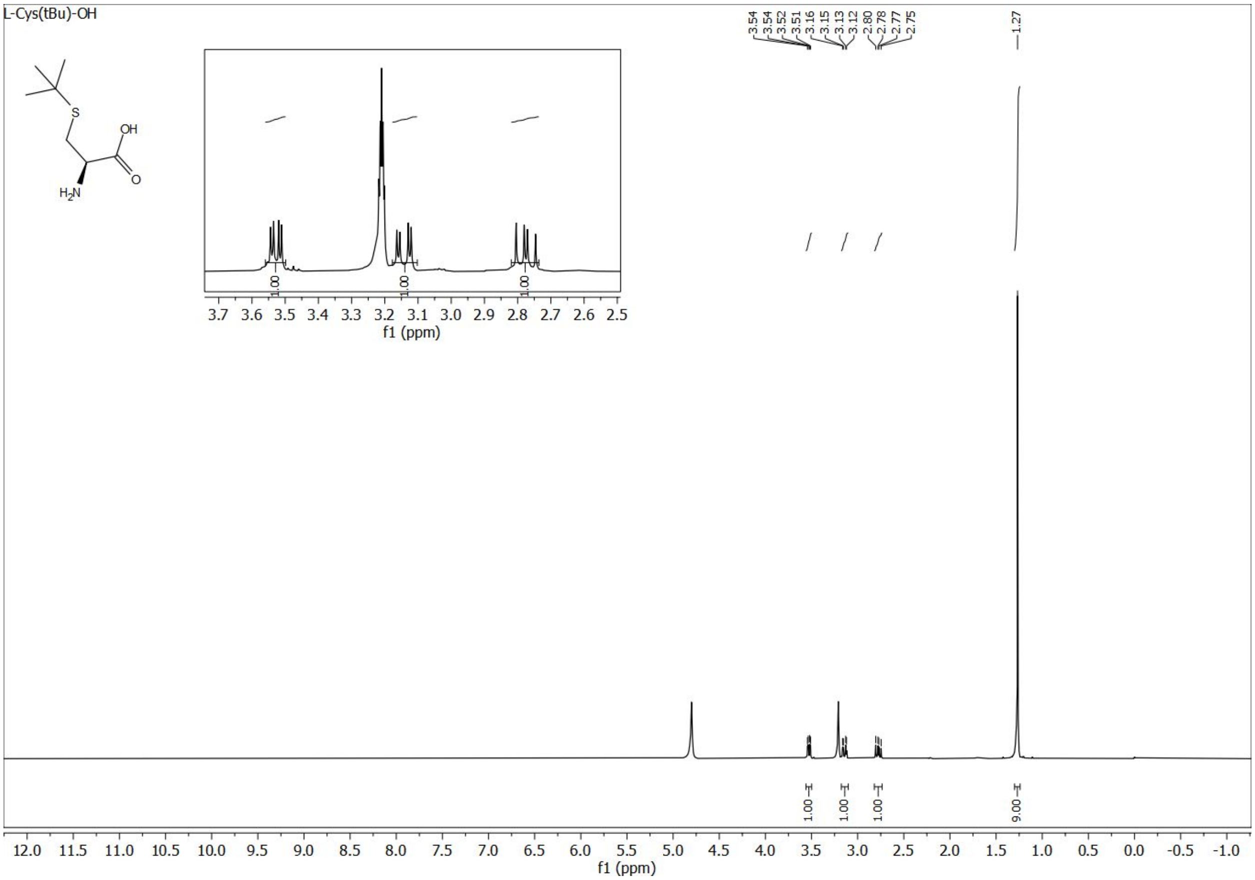

Figure S14: ^{1}H NMR spectrum of L-Cys(*t*Bu)-OH (400 MHz CD_3OD).

References

1. Postma, T. M.; Giraud, M.; Albericio, F. Trimethoxyphenylthio as a highly labile replacement for *tert*-butylthio cysteine protection in fmoc solid phase synthesis. Org. Lett. 2012,14, 5468.
2. Chakraborty, A.; Sharma, A.; Albericio, F.; de la Torre, B. G. Disulfide-based protecting groups for the cysteine side chain amit chakraborty. Org. Lett. 2020, 22, 9644.
